# A novel antimicrobial peptide acting via formyl peptide receptor 2 shows therapeutic effects against rheumatoid arthritis

**DOI:** 10.1038/s41598-018-32963-5

**Published:** 2018-10-02

**Authors:** Yoo Jung Park, Byunghyun Park, Mingyu Lee, Yu Sun Jeong, Ha Young Lee, Dong Hyun Sohn, Jason Jungsik Song, Joon Ha Lee, Jae Sam Hwang, Yoe-Sik Bae

**Affiliations:** 1 Department of Biological Sciences, Sungkyunkwan University, Suwon, 16419 Korea; 2 Department of Health Sciences and Technology, SAIHST, Sungkyunkwan University, Seoul, 06351 Korea; 3 Department of Microbiology and Immunology, Pusan National University School of Medicine, Yangsan, 50612 Korea; 4 Department of Internal Medicine, Division of Rheumatology, Yonsei University College of Medicine, Seoul, 03722 Korea; 5 Department of Agricultural Biology, National Academy of Agricultural Science, RDA, Wanju, 55365 Korea

## Abstract

In oriental medicine, centipede *Scolopendra subspinipes mutilans* has long been used as a remedy for rheumatoid arthritis (RA), a well-known chronic autoimmune disorder. However, the molecular identities of its bioactive components have not yet been extensively investigated. We sought to identify bioactive molecules that control RA with a centipede. A novel antimicrobial peptide (AMP) (scolopendrasin IX) was identified from *Scolopendra subspinipes mutilans*. Scolopendrasin IX markedly activated mouse neutrophils, by enhancing cytosolic calcium increase, chemotactic cellular migration, and generation of superoxide anion in neutrophils. As a target receptor for scolopendrasin IX, formyl peptide receptor (FPR)2 mediates neutrophil activation induced by the AMP. Furthermore, scolopendrasin IX administration strongly blocked the clinical phenotype of RA in an autoantibody-injected model. Mechanistically, the novel AMP inhibited inflammatory cytokine synthesis from the joints and neutrophil recruitment into the joint area. Collectively, we suggest that scolopendrasin IX is a novel potential therapeutic agent for the control of RA via FPR2.

## Introduction

Rheumatoid arthritis (RA), a well-known chronic autoimmune disorder, is characterized by synovial inflammation and autoantibody production^1^. During RA pathogenesis, several inflammatory immune cells, such as neutrophils, are recruited into the joint area and synovium^2^. The inflammatory cells contribute to inflammation and osteoclast differentiation, which leads to cartilage erosion and bone deformity^1,2^. Activated neutrophils play a crucial role in the modulation of the inflammatory response by communicating with other immune cells through the secretion of cytokines and chemokines^2^. Neutrophils also upregulate plasma membrane major histocompatibility complex (MHC)II in order to present antigen to T cells in RA pathophysiology^2^. Therefore, the identification of molecules that modulate neutrophil activity is critical to control RA.

Neutrophils express many different cell surface receptors including several chemokine/chemoattractant receptors^3^. Chemoattractant receptors such as formyl peptide receptor (FPR) mediate neutrophil recruitment into infected or injured areas^3,4^. Compared with other chemokine/chemoattractant receptors, FPRs recognize diverse ligands derived from the host, pathogens, or through artificial synthesis^4,5^. Recently, we also demonstrated several centipede-produced antimicrobial peptides (AMPs) that act on FPR members^6–8^. Historically, centipede *Scolopendra subspinipes mutilans* has long been used in oriental medicine as a remedy for RA^9,10^. Although centipede possesses bioactive components that can be used to control RA, their molecular identities have yet to be extensively investigated. Here, we discovered a novel AMP that stimulates FPR2 from *S. subspinipes mutilans*, and named it scolopendrasin IX (sequence: MCKYFIKIVSKSAKK-CONH_2_). We also found that the AMP shows therapeutic effects against RA by modulating cytokine production and neutrophil recruitment into the joint.

## Results

### A novel AMP that modulates neutrophil activity

**Figure 1:**
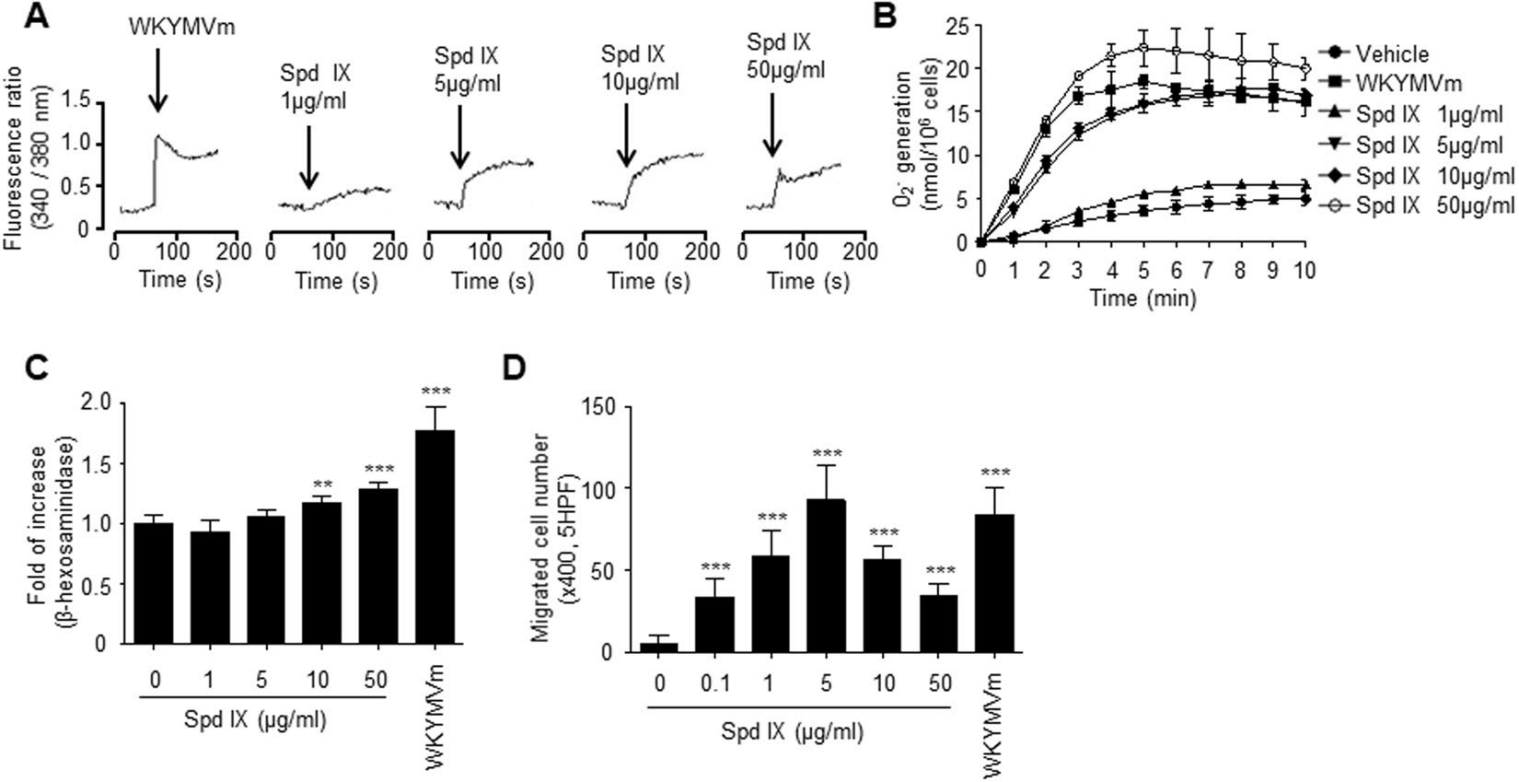
A novel AMP, scolopendrasin IX, stimulates neutrophils, resulting in calcium increase, superoxide anion production, and chemotactic migration. (**A**) Mouse neutrophils were treated with several concentrations (1 μg/ml, 5 μg/ml, 10 μg/ml, and 50 μg/ml) of scolopendrasin IX or WKYMVm (1 μM). Relative cytosolic Ca^2+^ concentrations are expressed as fluorescence ratios (340:380 nm). (**B**) Mouse neutrophils (1 × 10^6^ cells/100 μl of RPMI 1640 medium per well of a 96-well plate) were stimulated with different concentrations (0 μg/ml, 1 μg/ml, 5 μg/ml, 10 μg/ml, and 50 μg/ml) of scolopendrasin IX or 1 μM of WKYMVm. Superoxide anion production was determined by measuring cytochrome c reduction. (**C**) Mouse neutrophils (1 × 10^6^ cells) were resuspended in Tyrode’s buffer, and incubated with several concentrations (0 μg/ml, 1 μg/ml, 5 μg/ml, 10 μg/ml, and 50 μg/ml) of scolopendrasin IX or 1 μM of WKYMVm for 30 min. The peptide-induced secretion of β-hexosaminidase was determined. (**D**) Mouse neutrophils were applied to the upper well of a multi-well chamber containing different concentrations (0 μg/ml, 0.1 μg/ml, 1 μg/ml, 5 μg/ml, 10 μg/ml, and 50 μg/ml) of scolopendrasin IX or 1 μM of WKYMVm for 90 min. Data are presented as mean ± SD (n = 3 for B, C, n = 10 for **D**). The data are representative of three independent experiments (**A**). Data in panels are representative of two (**B**,**C**) or three (**D**) independent experiments. **p < 0.01, ***p < 0.001 compared with vehicle-treated control.

Neutrophils are one of the key players in the regulation of innate immunity to invading pathogens^11^. In this study, we tested the effects of several AMP candidates, which were identified from genome analysis of centipede *S. subspinipes mutilans* using a previously reported algorithm, on neutrophil activity^12^. Since neutrophil activation is associated with increased cytosolic calcium, we monitored cytosolic calcium levels following AMP stimulation. Of the tested AMPs, a novel AMP that is named scolopendrasin IX, elicited cytosolic calcium increase from mouse neutrophils (Fig. 1A and data not shown). Stimulation of mouse neutrophils with a novel AMP triggered the increase of cytosolic calcium concentration, showing concentration dependency with maximal activity at a peptide concentration of 10 μg/ml (Fig. 1A). Activated neutrophils generate superoxide anion, which is crucially used for the elimination of invading pathogens^13^. We found that the novel AMP strongly enhanced the generation of superoxide anion from mouse neutrophils. The amount of superoxide anion generated by the AMP at 10 μg/ml was similar to that of WKYMVm (Fig. 1B). Activated neutrophils also release granules such as β-hexosaminidase^14^. Here, we tested the effects of a novel peptide on degranulation. The novel AMP also strongly stimulated degranulation of neutrophils, showing concentration dependency (Fig. 1C).

Neutrophil recruitment is closely associated with the initiation of immune reaction in response to infection or injury^3,11^. In this study, we examined the effects of a novel AMP on neutrophil chemotactic migration. Scolopendrasin IX significantly stimulates neutrophil chemotactic migration, showing maximal activity at 5 μg/ml of the peptide (Fig. 1D). Scolopendrasin IX-induced neutrophil chemotaxis shows a bell-shaped concentration-response curve (Fig. 1D) similar to that previously shown by other chemoattractants^15,16^. Scolopendrasin IX induced neutrophil chemotactic migration at concentrations of 5 μg/ml is comparable to that of WKYMVm (Fig. 1D). Collectively, these results suggest that the newly identified novel AMP (scolopendrasin IX) modulates mouse neutrophil activity, causing cytosolic calcium increase, generation of superoxide anion, degranulation, and chemotactic migration of the cells.

### FPR2 mediates scolopendrasin IX-induced neutrophil activation

**Figure 2:**
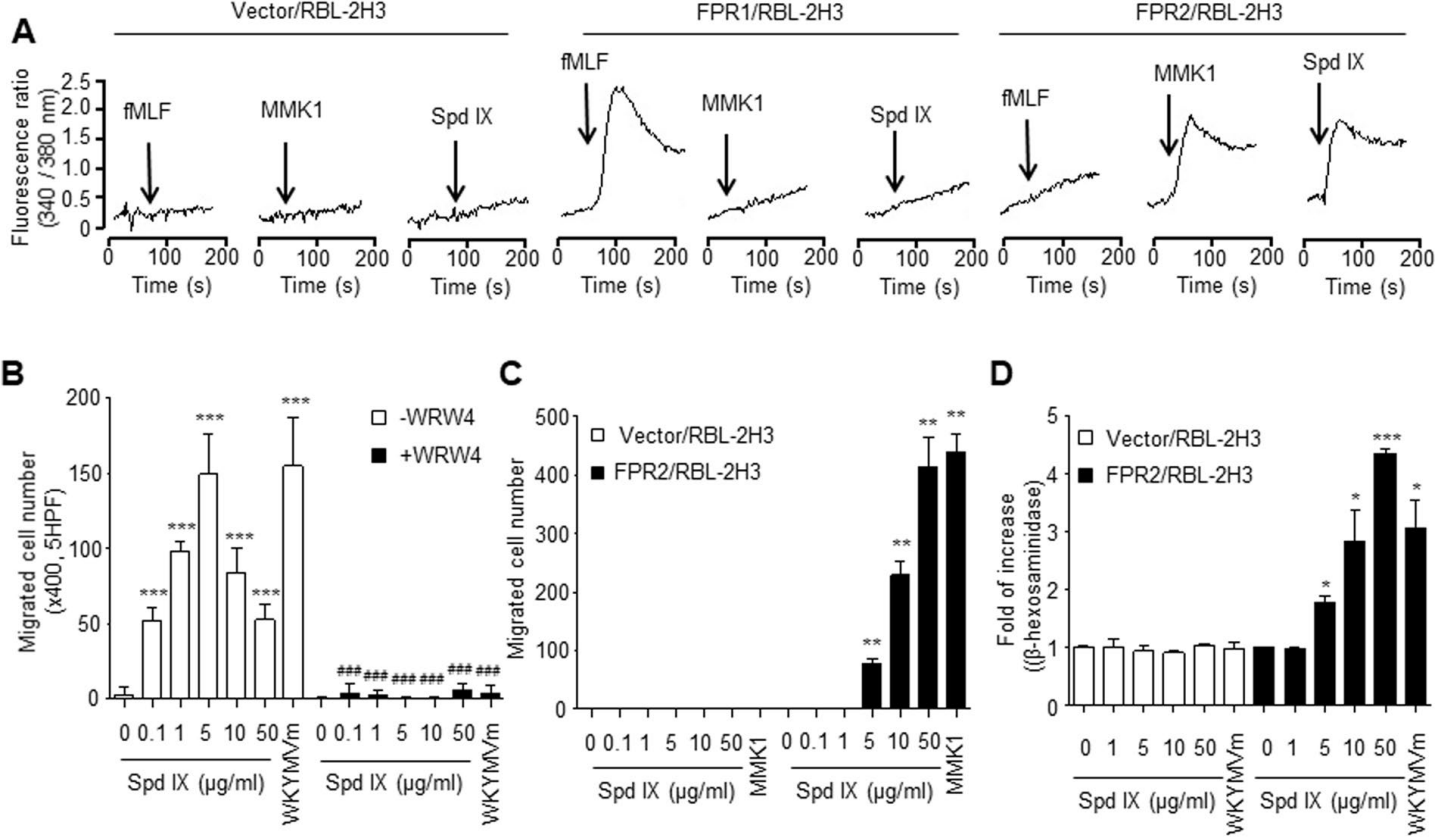
FPR2 mediates scolopendrasin IX-stimulated neutrophil activation. (**A**) Fura-2 loaded vector-, FPR1-, or FPR2-expressing RBL-2H3 cells were stimulated with 1 μM fMLF, 1 μM MMK-1, or 50 μg/ml scolopendrasin IX. Cytosolic calcium levels were determined fluorometrically using a spectrofluorophotometer. The peak levels of cytosolic calcium were recorded. (**B**) Neutrophils were pre-incubated with vehicle or WRW4 (40 μM) for 30 min, and then the cells were applied to the upper well of a multi-well chamber containing several concentrations (0 μg/ml, 0.1 μg/ml, 1 μg/ml, 5 μg/ml, 10 μg/ml, and 50 μg/ml) of scolopendrasin IX or WKYMVm (1 μM) for 90 min. The number of migrated cells was determined through counting under a light microscope. (**C**) Vector- or FPR2-expressing RBL-2H3 cells were applied to the upper well of a multi-well chamber containing several concentrations (0 μg/ml, 0.1 μg/ml, 1 μg/ml, 5 μg/ml, 10 μg/ml, and 50 μg/ml) of scolopendrasin IX or MMK-1 (1 μM) for 4 h. (**D**) Vector- or FPR2-expressing RBL-2H3 cells were re-suspended in Tyrode’s buffer and incubated with several concentrations (0 μg/ml, 1 μg/ml, 5 μg/ml, 10 μg/ml, and 50 μg/ml) of scolopendrasin IX or 1 μM of WKYMVm for 30 min. The peptide-induced secretion of β-hexosaminidase was determined. Data are presented as mean ± SD (n = 10 for **B**–**D**). Data are representative of two independent experiments (A). Data in panels are representative of two independent experiments (**B**–**D**). *p < 0.05, **p < 0.01, ***p < 0.001 compared with vehicle-treated control; ^###^*p* < 0.001 compared with control without WRW4 treatment.

Previously we reported that some novel AMPs, that were isolated from centipede *S. subspinipes mutilans*, act on FPR family members^6–8^. In this study, we examined whether scolopendrasin IX acted on the FPR family by measuring the effects of the AMP on the levels of cytosolic calcium concentration in RBL-2H3 cells, which express vector, FPR1, or FPR2, respectively. Addition of scolopendrasin IX markedly enhanced cytosolic calcium level in FPR2-expressing RBL-2H3 cells. However, the AMP did not change the cytosolic calcium concentration in vector or FPR1-expressing RBL-2H3 cells (Fig. 2A). Next, we tested whether the AMP stimulates neutrophil chemotactic migration via FPR2. Neutrophil chemotactic migration stimulated by scolopendrasin IX was almost completely blocked by an FPR2-selective antagonist WRW4 (Fig. 2B). We also found that scolopendrasin IX stimulated chemotactic migration of FPR2-expressing RBL-2H3 cells, showing concentration dependency (Fig. 2C). However, no significant change in cellular migration was observed by the AMP in vector-expressing RBL-2H3 cells (Fig. 2C). Selective migration of FPR2-expressing RBL-2H3 cells, but not the vector-expressing RBL-2H3 cells, was also induced by a previously known FPR2 agonist, MMK-1 (Fig. 2C). Based on the finding that scolopendrasin IX stimulated degranulation of neutrophils, we further investigated the role of FPR2 on AMP-induced degranulation. Scolopendrasin IX significantly stimulated degranulation in FPR2-expressing RBL-2H3 cells, and no effect was observed by the AMP in vector-expressing RBL-2H3 cells (Fig. 2D). In summary, our results show that the novel AMP scolopendrasin IX may act on FPR2, and then consequently elicits neutrophil activation which includes cytosolic calcium increase, chemotactic cellular migration, and degranulation.

### Scolopendrasin IX inhibits LPS-stimulated TNF-α, IL-6, and IL-10 synthesis in mouse neutrophils

**Figure 3:**
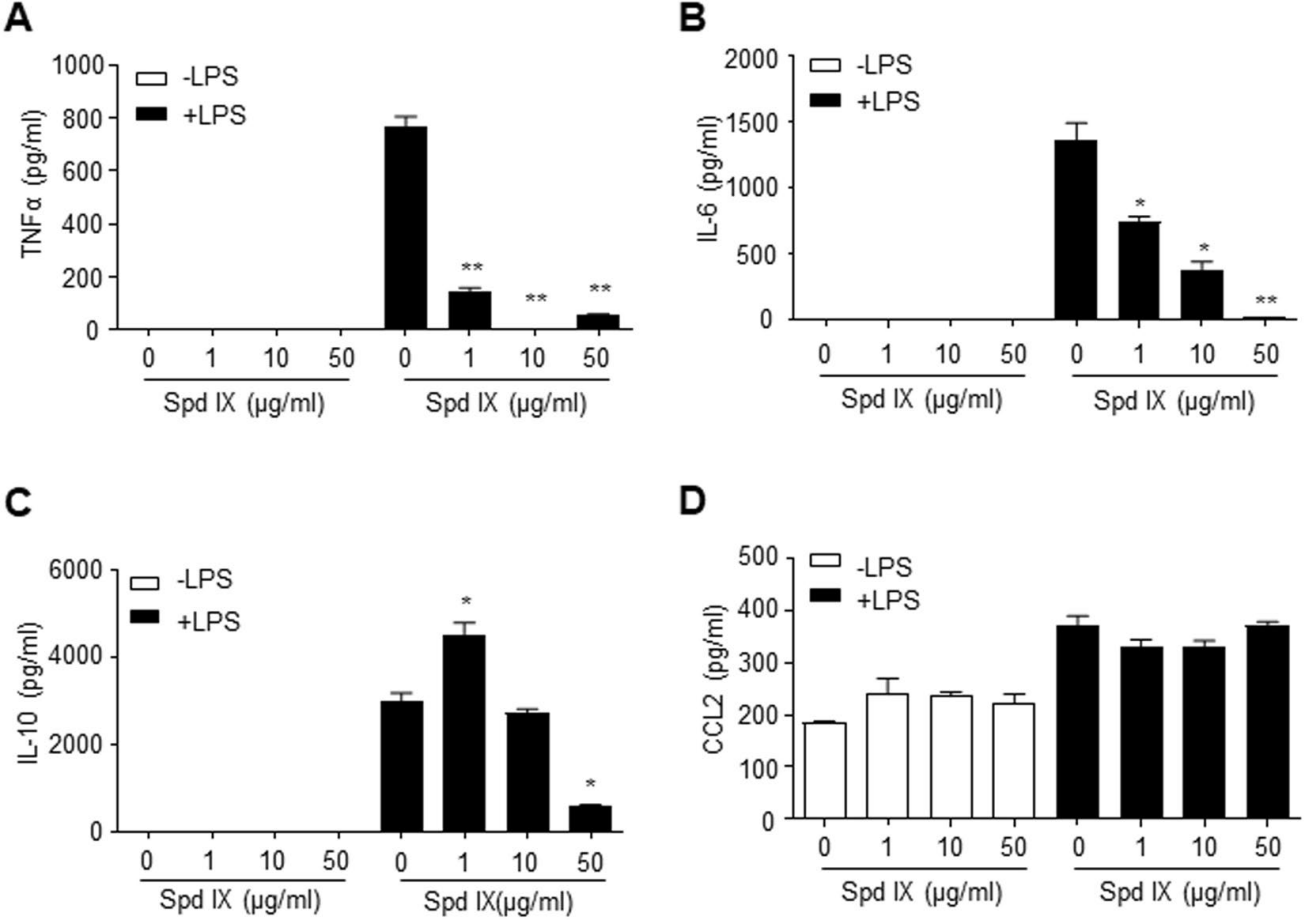
Scolopendrasin IX inhibits LPS-induced cytokine production in neutrophils. Mouse neutrophils were pre-incubated with vehicle (PBS) or scolopendrasin IX (0 μg/ml, 1 μg/ml, 10 μg/ml, and 50 μg/ml) for 30 min and stimulated with PBS or LPS (1 μg/ml) for 24 h. Levels of TNF-α (**A**) IL-6 (**B**) IL-10 (**C**) and CCL2 (**D**) were measured by ELISA. Data are presented as mean ± SD (n = 3 for A–D). Data in panels are representative of two independent experiments (A-D). *p < 0.05, **p < 0.01 compared with -LPS control.

In terms of the immunological role of FPR agonist, we previously demonstrated that FPR2 activation by an immune-modulating peptide WKYMVm suppresses LPS-induced inflammatory cytokine increase^17^. Our finding that scolopendrasin IX acted on FPR2 prompted us to investigate the regulatory role of AMP in cytokine production by using mouse neutrophils. Activation of mouse neutrophils with LPS markedly increased the levels of cytokines such as TNF-α, CCL2, IL-6, and IL-10 (Figs. 3A–D). LPS-induced production of TNF-α, IL-6, and IL-10 was significantly decreased by scolopendrasin IX in a concentration-dependent manner, showing maximal activity at 1 to 50 μg/ml (Figs. 3A–C). However, LPS-stimulated CCL2 production was not inhibited by scolopendrasin IX (Fig. 3D). In order to test whether the inhibitory effect of scolopendrasin IX on the production of cytokines induced by LPS is mediated by unexpected side effects, we tested the effects of scolopendrasin IX on toxicity in neutrophils. We found that the peptide did not induce cellular toxicity at 1~50 μg/ml (data not shown).

### Scolopendrasin IX shows therapeutic effects against RA

**Figure 4:**
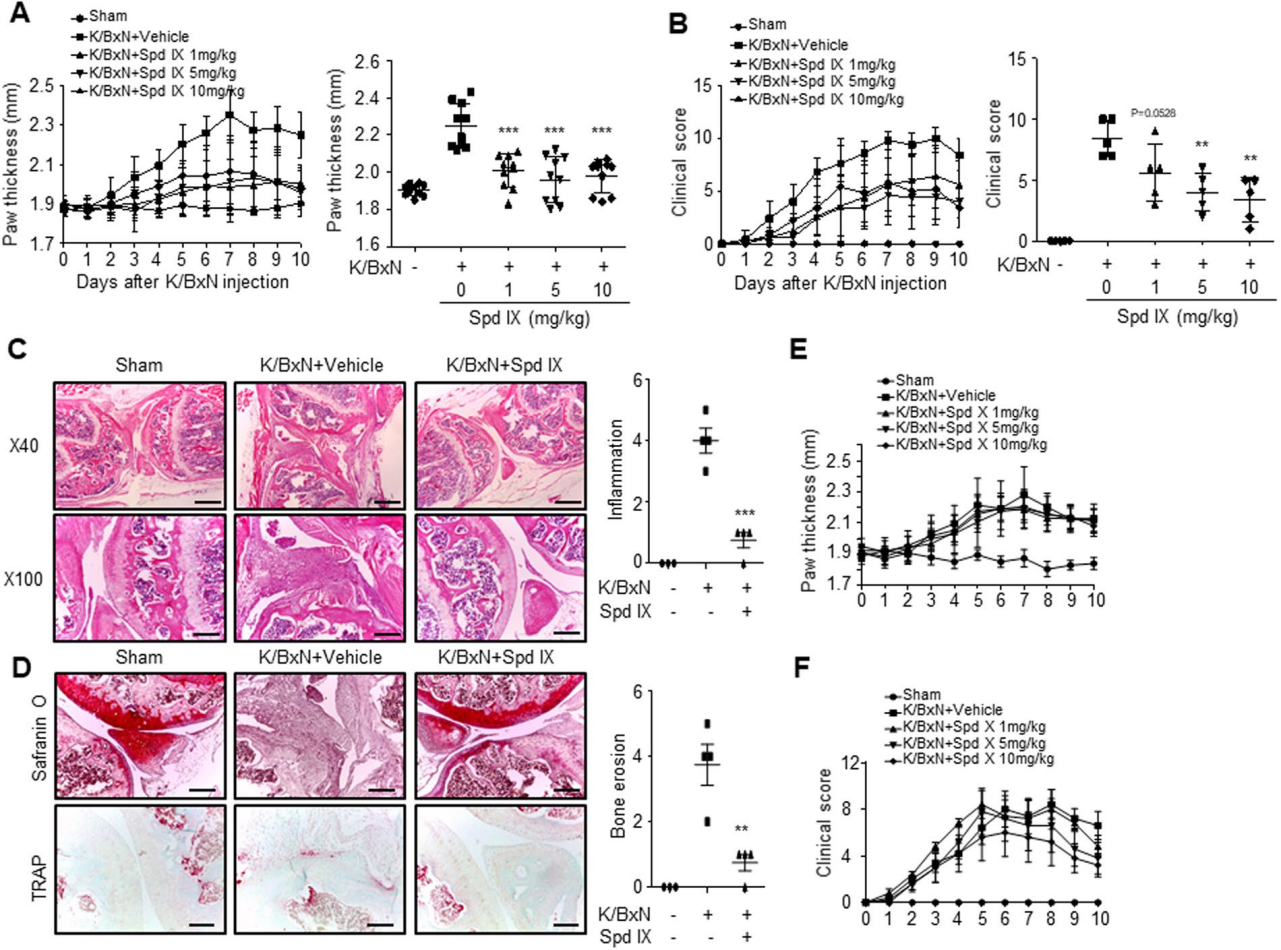
Administration of scolopendrasin IX induces therapeutic effects in the K/BxN serum-transfer arthritis model. Arthritis was induced in C57BL/6 mice by i.p. injection of K/BxN arthritogenic serum on days 0 and 2. (**A**) Paw thickness and (**B**) clinical score of C57BL/6 mice administrated with vehicle or different dosages of scolopendrasin IX (n = 5 mice per group) after K/BxN serum injection (left), and the measurements on day 10 were compared among each group (right). Paraffin tissue sections from sham and K/BxN serum-induced arthritic mice administrated with vehicle or 1 mg/kg scolopendrasin IX on day 10 were stained with (**C**) H&E (**D**) safranin O (×100), and TRAP (×100), shown under different magnifications. Arthritis was induced in C57BL/6 mice by i.p. injection of K/BxN arthritogenic serum on days 0 and 2. (**E**) Paw thickness and (**F**) clinical score of C57BL/6 mice administrated with vehicle or different dosages of scolopendrasin X (n = 5 mice per group) after K/BxN serum injection. Data are presented as mean ± SD (n = 5 for A, B, E, F, n = 3–4 for C right, D right). Data in panels are representative from two independent experiments (C left, D left). Scale bar, 500 μm (C ×40), 200 μm (C ×100, D). **p < 0.01, ***p < 0.001 compared with K/BxN alone control.

Centipedes have long been used to control RA in oriental medicine^9,10^. We found that a novel AMP, scolopendrasin IX, suppresses inflammatory cytokine levels elicited by LPS stimulation in mouse neutrophils. In our study, we tested the effects of the AMP on the K/BxN serum-transfer arthritis model. Administration of serum from the arthritic transgenic K/BxN mice to the C57BL/6 mice induced peripheral joint inflammation manifesting as increased paw thickness (Fig. 4A). The administration of scolopendrasin IX in K/BxN serum-injected mice significantly decreased paw thickness in a dose-dependent manner. The therapeutic effects of scolopendrasin IX against inflammatory arthritis were apparent at 1 mg/kg to 10 mg/kg (Fig. 4A). The clinical score of inflammatory arthritis was also markedly decreased by scolopendrasin IX at 1 mg/kg to 10 mg/kg (Fig. 4B).

RA progression is associated with joint destruction^1,2^. In this study, we also found that injection of the K/BxN serum induced cartilage destruction and inflammatory cell recruitment into the synovium (Fig. 4C). The administration of scolopendrasin IX markedly ameliorated joint destruction induced by K/BxN serum injection (Fig. 4C). The joint remained intact following scolopendrasin IX administration in K/BxN serum-injected mice (Fig. 4C). Inflammatory cell recruitment into the synovium was also strongly inhibited by scolopendrasin IX administration (Fig. 4C). Since joint destruction is associated with increased osteoclast activity^18^, we investigated the effects of scolopendrasin IX on osteoclast activity using safranin O staining and TRAP staining. The injection of K/BxN strongly induced a loss of articular cartilage, reflected by reduced safranin O staining and increased TRAP staining (Fig. 4D). However, the administration of scolopendrasin IX markedly restored articular cartilage with intact safranin O staining in K/BxN serum-injected mice (Fig. 4D). TRAP-positive cells in joint cartilage were also strongly decreased by scolopendrasin IX administration (Fig. 4D). These results suggest that scolopendrasin IX was therapeutically effective against inflammatory arthritis by blocking joint destruction and restoring articular cartilage. Since we previously reported another AMP, scolopendrasin X, derived from *Scolopendra subspinipes mutilans*^8^, we have tested if scolopendrasin X has therapeutic effects in a K/BxN serum transfer model. However, we could not observe any therapeutic activity of the peptide in the RA model (Figs. 4E and 4F). These results suggest that the therapeutic effects of scolopendrasin peptide are specific for scolopendrasin IX but not scolopendrasin X.

### Scolopendrasin IX blocks neutrophil infiltration into joint and inflammatory cytokine production in the K/BxN serum-transfer arthritis model

**Figure 5:**
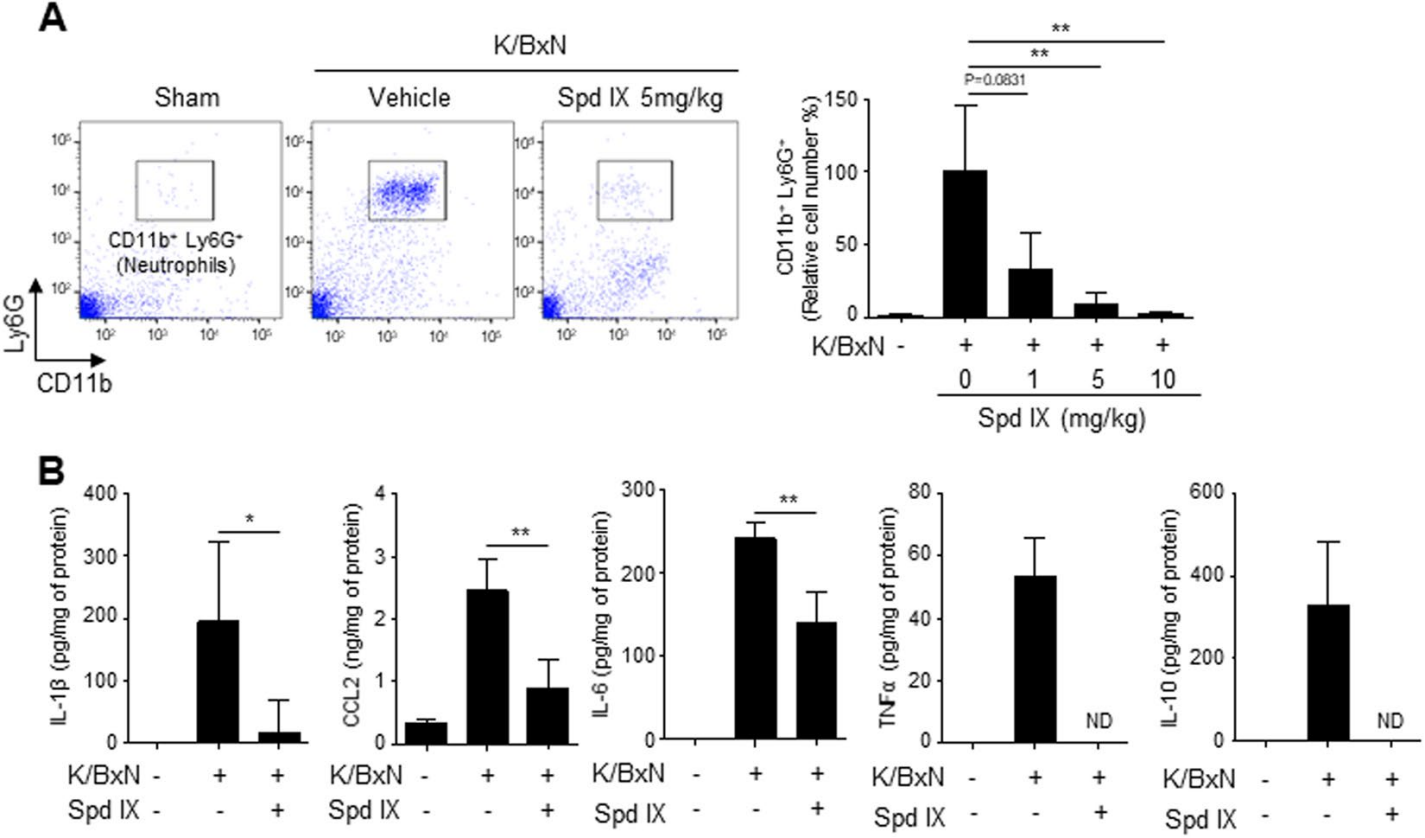
Scolopendrasin IX inhibits inflammatory cytokine production as well as neutrophil recruitment into the synovium joint area in the K/BxN serum-transfer arthritis model. (**A**) On day 10 after initiation of arthritis, cells in the synovial fluid from each group were stained with anti-CD11b and anti-Ly6G, and neutrophils (CD11b^+^, Ly6G^+^) were analyzed by flow cytometry (left). Data are shown in several concentrations (0 mg/kg, 1 mg/kg, 5 mg/kg, 10 mg/kg) of scolopendrasin IX (right). (**B**) Ankle joint tissues from sham and K/BxN serum-induced arthritic mice injected with vehicle or scolopendrasin IX (1 mg/kg) were homogenized on day 10, and the cytokine levels were measured by ELISA. Data are presented as mean ± SD (n = 5 for A right, **B**). Data are representative of five mice per group (A left). Data in panels are representative from two independent experiments (A right, B). *p < 0.05, **p < 0.01.

During RA progression, neutrophils are recruited into the joint area and subsequently contribute to the inflammatory response^2,3^. In this study, we also found that K/BxN serum injection induced neutrophil infiltration into the joint area, which was analyzed through flow cytometry of the synovial fluid (Fig. 5A). We then tested the effects of scolopendrasin IX on neutrophil recruitment into the joint area in K/BxN serum-injected mice. The administration of scolopendrasin IX strongly blocked neutrophil infiltration induced by the K/BxN serum (Fig. 5A, left). The inhibitory effects of scolopendrasin IX on neutrophil recruitment were apparent at 1 to 10 mg/kg (Fig. 5A, right). Near complete inhibition of neutrophil recruitment in the K/BxN serum-transfer arthritis model was observed at 10 mg/kg of scolopendrasin IX (Fig. 5A right).

RA progression induces the synthesis of several inflammatory cytokines in the joint area^1,2,18^. We also found that the K/BxN serum injection increased the production of inflammatory cytokines such as IL-1β, CCL2, IL-6, TNF-α, and IL-10 in the joint (Fig. 5B). However, the administration of scolopendrasin IX significantly decreased the production of these inflammatory cytokines as well as of IL-10 in the joints of K/BxN serum-injected mice (Fig. 5B). These results suggest that scolopendrasin IX was therapeutically effective against inflammatory arthritis by blocking neutrophil recruitment into the joint area, and by subsequently inhibiting inflammatory cytokine production.

### FPR2 mediates scolopendrasin IX-induced therapeutic effects against K/BxN serum-transfer arthritis

**Figure 6:**
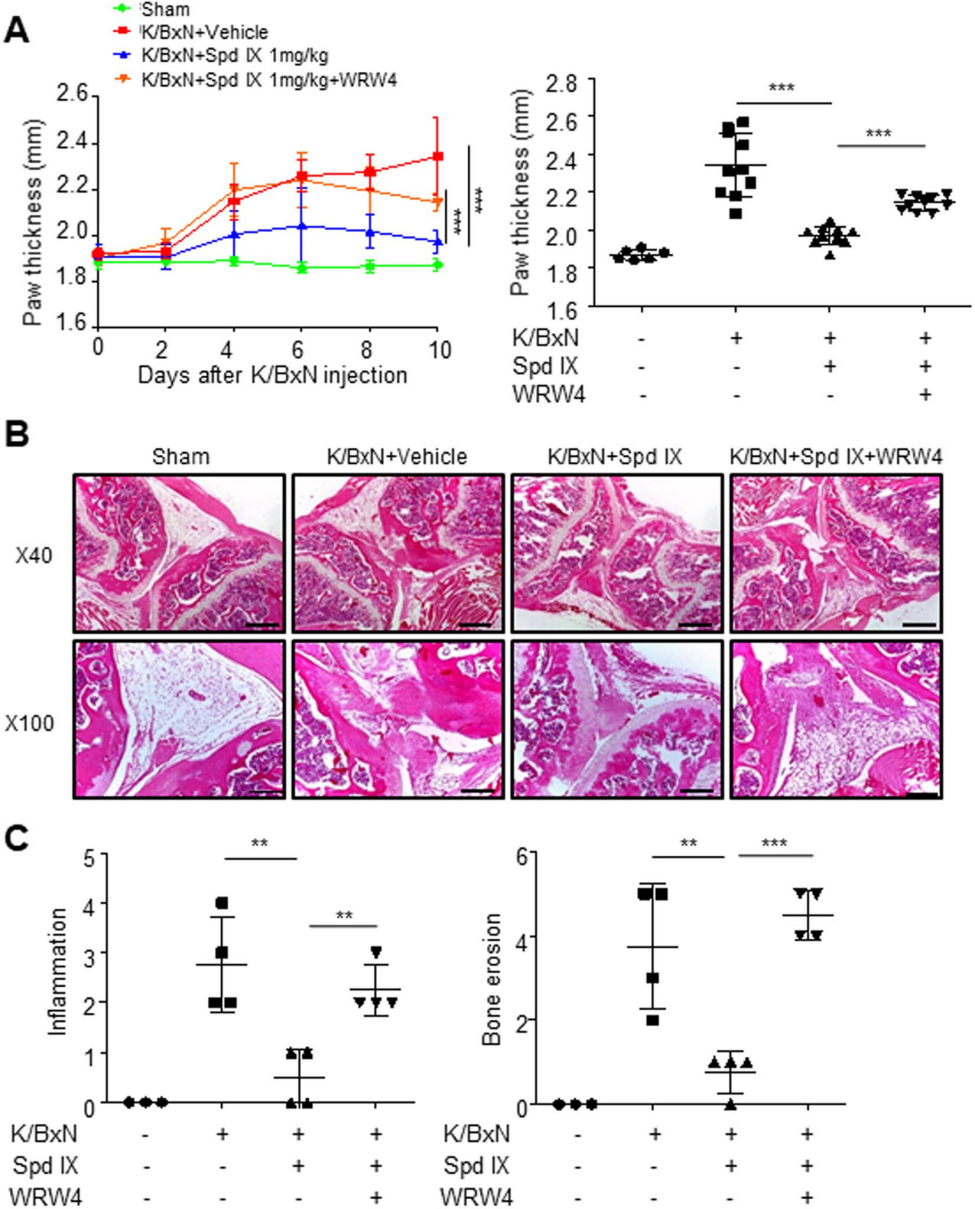
WRW4 inhibits scolopendrasin IX-induced inhibitory effects against K/BxN serum-transfer arthritis. Arthritis was induced in C57BL/6 mice by i.p. injection of K/BxN arthritogenic serum on days 0 and 2. (**A**) The paw thickness of C57BL/6 mice administrated with vehicle, scolopendrasin IX (1 mg/kg), or WRW4 (1 mg/kg) + scolopendrasin IX (1 mg/kg) (n = 5 mice per group) after K/BxN serum injection (left), and measurements on day 10 were compared among each group (right). (**B**) Paraffin tissue sections from sham and K/BxN serum-induced arthritic mice administrated with vehicle, scolopendrasin IX (1 mg/kg), or WRW4 (1 mg/kg) + scolopendrasin IX (1 mg/kg) on day 10 were stained with H&E shown under different magnifications (×40 and ×100). (**C**) Histological scores of inflammation and bone erosion are quantified. Data are presented as mean ± SD (n = 5 for A, n = 3–4 for **C**). Data in panels are representative of three or four mice per group from two independent experiments (**B**). Scale bar, 500 μm (B ×40), 200 μm (B ×100). **p < 0.01, ***p < 0.001.

Based on the finding that scolopendrasin IX acts on FPR2 and the peptides show anti-arthritic effects in the K/BxN serum transfer model, we investigated the role of FPR2 in mediating the therapeutic activity of the peptide against RA using an FPR2-selective antagonist, WRWWWW (WRW4)^19^. Administration of WRW4 prior to scolopendrasin IX in the K/BxN serum transfer RA model significantly blocked the beneficial effects of scolopendrasin IX (Fig. 6A). Scolopendrasin IX decreased paw thickness in the serum transfer model, which was markedly blocked by WRW4 administration (Fig. 6A). The inhibitory effects of scolopendrasin IX on cartilage destruction in the K/BxN serum transfer model was also markedly blocked by WRW4 (Fig. 6B). Quantitative analyses showed that scolopendrasin IX also significantly attenuated cartilage inflammation and bone erosion in the K/BxN serum transfer model, which were also strongly blocked by the administration of WRW4 (Fig. 6C). The results suggest that FPR2 is an important factor in scolopendrasin IX-induced anti-arthritic activity.

## Discussion

We have discovered a novel AMP which stimulates neutrophil activity from *Scolopendra subspinipes mutilans*, and named it scolopendrasin IX. In terms of functional aspect, scolopendrasin IX increased cytosolic calcium level, superoxide anion production, degranulation, and chemotactic migration. In addition, this AMP strongly suppressed LPS-induced inflammatory cytokine levels in neutrophils. We also observed that the AMP acted on FPR2. Using the RA disease model, we demonstrated that the administration of scolopendrasin IX induced therapeutic effects against RA. According to our results, we suggest that the novel AMP (scolopendrasin IX) can be regarded as a potential therapeutic candidate for RA treatment.

Several reports have previously demonstrated that FPR2 can be considered as an important target molecule to control human disease^5,20^. The activation of FPR2 using selective agonists has been shown to induce therapeutic effects for several infectious- or inflammatory diseases such as polymicrobial sepsis, ulcerative colitis, and certain cancers^17,21,22^. Here, we found that the administration of scolopendrasin IX elicited intense therapeutic effects in the K/BxN serum-transfer arthritis model. Administration of the peptide ameliorated arthritis severity, showing decreased paw thickness and clinical scores (Figs. 4A and 4B). Joint damage was also markedly blocked by the AMP, which was accompanied by undamaged proteoglycans in the K/BxN serum-transfer arthritis model (Figs. 4C and 4D). Cartilage damage in the K/BxN serum-transfer arthritis model is mediated by the activation of osteoclasts^23,24^. Our findings on the inhibitory effects of scolopendrasin IX against K/BxN serum-induced cartilage damage suggest that the administration of the novel peptide blocked osteoclast differentiation and activation of matrix-metalloproteinase. Our findings suggest that scolopendrasin IX targeted FPR2, and that the therapeutic effects against inflammatory arthritis, and recovery of proteoglycan induced by the peptide are mediated via FPR2. The functional roles of FPR2 and its ligands on RA are controversial^25–28^. Previously, treatment of cpd43 (a dual agonist of FPR1 and FPR2) has been reported to decrease clinical severity in the K/BxN serum-transfer arthritis model^25^. A mimic of annexin A1, superAnnexin A1, has also been reported to induce anti-arthritic effects^26^. Deletion of annexin A1 (an endogenous FPR2 agonist) has been shown to exacerbate arthritis severity in the K/BxN serum-injected mice^25,27^, suggesting a crucial role of annexin A1 in the regulation of RA pathogenesis. However, human annexin A1 has been reported to induce increased clinical signs of RA in the collagen-induced arthritis model by modulating T cell differentiation^28^. Here, we showed that a novel FPR2 agonist (scolopendrasin IX) elicited strong therapeutic effects against the K/BxN serum-transfer arthritis model (Fig. 4), which was blocked by an FPR2 antagonist (WRW4) (Fig. 6), suggesting that stimulation of FPR2 resulted in therapeutic activity against RA.

In parallel experiments, we found that the administration of another agonist, scolopendrasin X, failed to induce therapeutic effects in the K/BxN serum-transfer arthritis model (Figs. 4E and 4F). These results suggest ligand-selective therapeutic effects of FPR2 agonists against inflammatory arthritis. Previous studies have demonstrated that an important chemoattractant receptor FPR2 can be differentially activated by different agonists in a ligand-selective manner^16,29–31^. Stimulation of FPR2 by its different agonists such as synthetic peptides, WKYMVm and its analogues, HEYLPM and HRYLPM, serum amyloid A and WKYMVm, and lipoxin A4 and MMK-1 elicited differential downstream signaling in FPR2-expressing cells^16,29–31^. The two scolopendrasin peptides, scolopendrasin X^8^ and scolopendrasin IX, showed the same cellular signaling and cellular responses including the activation of cytosolic calcium increase, superoxide anion production, chemotactic migration, and inhibition of inflammatory cytokines from mouse neutrophils (Figs. 1–3). However, scolopendrasin IX but not scolopendrasin X markedly decreased inflammatory cytokine production in the K/BxN serum transfer model (Figs. 4 and 5). It is currently unclear what causes the differential effects of the two different AMPs on the experimental arthritis model. The detailed mechanisms of the actions of the two AMPs should be studied *in vivo* in the future.

We demonstrated that administration of the peptide scolopendrasin IX strongly decreased inflammatory cytokine production and subsequent neutrophil recruitment into synovium in the K/BxN serum-transfer arthritis model (Figs. 5A and 5B). Scolopendrasin IX significantly blocked the synthesis of proinflammatory cytokines such as IL-1β, CCL2, and IL-6 from the synovial joints of arthritic mice (Fig. 5B). We previously showed that FPR2 signaling activated by its selective synthetic agonist WKYMVm inhibited the levels of inflammatory cytokine caused by LPS and in models of cecal ligation and puncture sepsis^17^, suggesting that activation of FPR2 negatively regulates Toll-like receptor 4 signaling via cross-talk between FPR2 and Toll-like receptor 4. Our findings of the inhibitory effects of scolopendrasin IX on the production of inflammatory cytokines in response to K/BxN serum injection suggest that autoantibody-induced inflammatory response is negatively regulated by FPR2 activation, and that a novel FPR2 agonist elicits an anti-inflammatory response in the K/BxN serum-transfer arthritis model. Here, we also showed that scolopendrasin IX strongly inhibited neutrophil recruitment into the synovium following K/BxN serum injection (Fig. 5A). Previously, it was reported that activated neutrophils produce IL-1β in synovial fluid^32^. IL-1β stimulates chemokine synthesis in synovial endothelial cells, fibroblast-like synoviocytes, and macrophages^32^. Beyond that, IL-1β produced from neutrophil has a synergistic effect on RANKL-induced osteoclast differentiation and can activate mature osteoclast through NF-κB signaling^33^. Our data showed that scolopendrasin IX could suppress the production of IL-1β in joint tissue (Fig. 5B), which can explain the decrease of cartilage damage in the synovium caused by the administration of scolopendrasin IX (Fig. 4D). We also found that scolopendrasin IX significantly decreased the production of the chemokine CCL2 (Fig. 5B). These results suggest that the therapeutic activity of scolopendrasin IX in the K/BxN serum-transfer arthritis model was mediated via the inhibition of cytokine-chemokine cascade and by blocking neutrophil infiltration into the joint.

In conclusion, we have identified a novel AMP peptide with therapeutic effects in the K/BxN serum-transfer arthritis model, a well-known animal model of RA. Mechanistically, the novel AMP blocked K/BxN serum-induced inflammatory cytokine production and neutrophil recruitment into the joint area. Our findings suggest that the peptide, scolopendrasin IX, represents an important therapeutic candidate in the management of autoimmune arthritis.

## Materials and Methods

### Materials

Synthetic peptides such as scolopendrasin IX, WKYMVm, MMK-1, and WRW4 were provided by Anygen (Gwangju, Korea). The purity of all the synthetic peptides was >99.6%. fMLF and 4-nitrophenyl N-acetyl-β-D-glucosaminide were obtained from Sigma-Aldrich (St. Louis, MO, USA). Chemotaxis assay chamber (Boyden chambers) were provided from Neuroprobe, Inc. (Gaithersburg, MD, USA). Fura-2 penta-acetoxymethylester (fura-2/AM) was purchased from Molecular Probes (Eugene, OR, USA). RPMI 1640 and HBSS-EDTA were provided by Welgene (Gyeongsan, Korea).

### Enrichment of mouse neutrophils

All animal experiments were performed in accordance with the guidelines of the Korea Food and Drug Administration. All experiments involving animals received the approval of the Institutional Review Committee for Animal Care and Use at Sungkyunkwan University (Suwon, Korea). Mouse bone marrow neutrophil isolation was conducted according to a previous report^34^. The purity of isolated mouse bone marrow neutrophils was tested by staining the cells with fluorescence containing anti-Ly6G antibody and subsequent analysis using flow cytometry (BD FACSCanto II, Franklin Lakes, NJ, USA), which showed more than 95% Ly6G-positive cells.

### Measurement of cytosolic calcium levels

The level of cytosolic calcium concentration was measured according to a previous report^35^. At first, purified mouse bone marrow neutrophils, and cultured vector-, FPR1-, or FPR2- expressing RBL-2H3 cells were incubated with fura-2/AM (3 μM) at 37 °C for 50 min. Fura-2/AM loaded cells were aliquoted at 1 × 10^7^ in Locke’s solution (154 mM NaCl, 5.6 mM KCl, 1.2 mM MgCl_2_, 5 mM HEPES, pH 7.3, 10 mM glucose, 2.2 mM CaCl_2_, and 0.2 mM EGTA). Following specific stimulation, the changes in fluorescence ratios (340 nm vs 380 nm) were monitored using a spectrofluorophotometer (RF5301PC, SHIMADZU, Tokyo, Japan).

### Measurement of superoxide anion production

The levels of superoxide anion were measured as previously described^36^. At first, purified mouse neutrophils were suspended in 100 μl of RPMI 1640 medium at 1 × 10^6^ cells density, and subsequently incubated with cytochrome c (50 μM) and cytochalasin B (5 μM) for 5 min. Then, different stimuli were given to the cells for 10 min. Superoxide generation was measured by monitoring the changes in light absorption at 550 nm by using a microtiter 96-well plate ELISA reader (Bio-Tek instruments, EL312e, Winooski, VT, USA).

### Degranulation assay

Degranulation was measured using a β-hexosaminidase assay, as previously described^29^. Purified mouse neutrophils and cultured vector-, or FPR2- expressing RBL-2H3 cells in Tyrode’s buffer (137 mM NaCl, 12 mM NaHCO_3_, 5.6 mM glucose, 2.7 mM KCl, 1 mM CaCl_2_, 0.5 mM MgCl_2_, 0.4 mM NaH_2_PO_4_, 0.1 g/100 ml BSA, 25 mM HEPES, pH 7.4) were applied with different stimuli at 37 °C and 5% CO_2_ for 30 min. The supernatant and lysed cells in the lysis buffer (0.6% Triton X-100 in PBS) were reacted with the substrate solution (5 mM 4-nitrophenyl N-acetyl-β-D-glucosaminide in 0.1 M citrate buffer, pH 3.8) at 37 °C and 5% CO_2_ for 2 h. Next, the stop solution (0.4 M Na_2_CO_3_) was added in order to stop the reaction, and OD 405 nm values were measured using a spectrophotometer.

### Chemotaxis assay

Chemotaxis assays were carried out according to previous reports^37,38^. Purified mouse bone marrow neutrophils and cultured vector-, FPR1-, or FPR2-expressing RBL-2H3 cells applied to Boyden chamber with polycarbonate filters (3 μm pore for mouse neutrophils, 8 μm pore for RBL-2H3 cells) at 37 °C. After 90 min (for mouse neutrophils) or 4 h (for RBL-2H3 cells), non-migrated cells were discarded from filter. The migrated cells were counted by using a light microscope, as previously described^37,38^.

### Measurement of cytokines

Mouse neutrophils (5 × 10^5^ cells/500 μl of 2% FBS containing RPMI 1640 medium) were stimulated with LPS (1 μg/ml) for 24 h. To find out the effects of scolopendrasin IX on the LPS-stimulated cytokine production, scolopendrasin IX was applied before LPS stimulation. The levels of cytokines were measured from the cell-free supernatants by ELISA (Thermo Fisher Scientific, Waltham, MA, USA).

### K/BxN serum transfer arthritis model

Male C57BL/6 mice aged more than eight weeks (purchased from Orient Bio Inc. (Seongnam, Korea)) were used for all experiments. KRN T cell receptor transgenic mice with a B6 background were bred to NOD mice in order to generate K/BxN mice expressing the T cell receptor transgene KRN and MHC class II molecule I-Ag7. The K/BxN serum was collected from arthritic K/BxN offspring and the serum diluted 50% in PBS was transferred into B6 mice through 100 μl i.p. injections on days 0 and 2, as previously described^39^. Vehicle or scolopendrasin IX were subcutaneously injected daily for 10 days into K/BxN serum transferred mice. Paw thickness and clinical score were monitored daily for up to 10 days.

### Histology of arthritic joints

Mice were sacrificed on day 10, and their knee joints were fixed in 4% paraformaldehyde solution. Fixed joints were decalcified in 10% formic acid for five days and embedded in paraffin. Joint tissues were sectioned by microtome and stained with hematoxylin & eosin for morphological analysis, with safranin O for the determination of cartilage damage, or with tartrate-resistant acid phosphatase (TRAP) in order to grade osteoclast formation.

### Flow cytometry analysis

Synovial fluid was collected from the ankle of each group of K/BxN arthritic mice injected with vehicle or scolopendrasin IX. Cells in the synovial fluid were collected by centrifugation of 300 g for 10 min and stained with anti-Ly6G and anti-CD11b antibody. Neutrophils recruited into the joint cavity were analyzed using flow cytometry.

### Measurement of cytokines *in vivo*

To determine the cytokine levels expressed, the ankle joint tissue was collected from each K/BxN serum-transferred mouse injected with vehicle or scolopendrasin IX on day 10. Ankle joint tissues were frozen and pulverized in liquid nitrogen. Frozen ankle joint tissues were homogenized in RIPA buffer (leupeptin 1 μg/ml, NaF 10 mM, pepstatin 1 μg/ml, PMSF 1 mM, and aprotinin 1 μg/ml) and the homogenized samples were centrifuged at 12,000 rpm for 10 min at 4 °C. The cytokines in the supernatant were measured by ELISA (Thermo Fisher Scientific, Waltham, MA, USA).

### Data analysis

Results were expressed as mean ± SD. Student’s *t*-test or ANOVA test was used to compare individual treatments with their respective control values. Statistical significance was set at *p* < 0.05.
